# The Strawberry Leaf—A Valuable Auxiliary in the Treatment of Chronic Dysentery

**Published:** 1848-01

**Authors:** 


*The Strawberry Leaf—A Valuable Auxiliary in the Treatment of Chronic Dysentery.* By J. C. C. Blackburn, M. D., of Barnesville, Georgia.

Believing that a discovery, however simple, which has a tendency to alleviate the sufferings of man, should be given without reserve to the medical world, I feel disposed to present to its consideration the claims of the Wild Strawberry. For the last three years I have been endeavoring to analyze this plant, and to try, if possible, to arrive at its medicinal properties. I was led to this investigation from the mere casual fact of seeing a dog, that was apparently in severe pain, swallowing its leaves. And here just let me add, that if physicians would more frequently lend an observing eye to the conduct of the brute creation, when afflicted with diseases peculiar to them, they might find remedies for diseases, which though at present obtainable, yet remain undiscovered. I have used the strawberry leaves in every form, for the cure of dysentery; but the formula most desirable is as follows: ℞. one pound of the green leaves, add to them one quart of good French brandy, and boil to one pint. Give of the strained liquor one table-spoonful every three hours, until the disease in question be relieved of its distressing symptoms. I will here add *one* case, of the origin of which I am totally ignorant.

Mr. B., a volunteer returned from Mexico, was taken with dysentary at Matamoras last August a year ago. He was placed under the direction of the Surgeon to the Georgia Regiment, who attended him until he pronounced his case incurable. The patient afterwards recovered sufficient strength to accompany the regiment to Monterey, and thence to Vera Cruz, where he was again prostrated by this disease. He reached home last July, with a constitution almost broken down, and placed himself under my care. I resorted to the use of every agent within my knowledge for the cure of his disease, but without success. I at length determined to try the strawberry leaves, as in the formula above-mentioned. He had taken but ten spoonfuls when he commenced to improve, and speedily recovered. He is now *entirely cured*, and able to attend to the duties of his calling. I have used the strawbery leaves in many cases since, with the same happy result.—*Southern Medical and Surgical Journal.*

